# The changing health effects of air pollution exposure for respiratory diseases: a multicity study during 2017–2022

**DOI:** 10.1186/s12940-024-01083-1

**Published:** 2024-04-13

**Authors:** Siyu Jiang, Longjuan Tang, Zhe Lou, Haowei Wang, Ling Huang, Wei Zhao, Qingqing Wang, Ruiyun Li, Zhen Ding

**Affiliations:** 1 School of Public Health, Nanjing Medical University, 101 Longmian AV, Nanjing, 211166 Jiangsu China; 2 School of Public Health, Imperial College London, London, UK; 3 MRC Centre for Global Infectious Disease Analysis and Abdul Latif Jameel Institute for Disease and Emergency Analytics, Imperial College London, London, UK; 4 College of Urban and Environmental Sciences, Peking University, Beijing, China; 5 Jiangsu Provincial Center for Disease Prevention and Control, 172 Jiangsu Rd, Nanjing, 210009 Jiangsu China; 6 Jiangsu Center for Collaborative Innovation in Geographical Information Resource Development and Application, Nanjing, China

**Keywords:** Air pollution, Respiratory disease, Health risks, Jiangsu, Epidemiology

## Abstract

**Background:**

Multifaceted SARS-CoV-2 interventions have modified exposure to air pollution and dynamics of respiratory diseases. Identifying the most vulnerable individuals requires effort to build a complete picture of the dynamic health effects of air pollution exposure, accounting for disparities across population subgroups.

**Methods:**

We use generalized additive model to assess the likely changes in the hospitalisation and mortality rate as a result of exposure to PM2.5 and O_3_ over the course of COVID-19 pandemic. We further disaggregate the population into detailed age categories and illustrate a shifting age profile of high-risk population groups. Additionally, we apply multivariable logistic regression to integrate demographic, socioeconomic and climatic characteristics with the pollution-related excess risk.

**Results:**

Overall, a total of 1,051,893 hospital admissions and 34,954 mortality for respiratory disease are recorded. The findings demonstrate a transition in the association between air pollutants and hospitalisation rates over time. For every 10 µg/m^3^ increase of PM2.5, the rate of hospital admission increased by 0.2% (95% CI: 0.1–0.7%) and 1.4% (1.0–1.7%) in the pre-pandemic and dynamic zero-COVID stage, respectively. Conversely, O_3_-related hospitalization rate would be increased by 0.7% (0.5–0.9%) in the pre-pandemic stage but lowered to 1.7% (1.5–1.9%) in the dynamic zero-COVID stage. Further assessment indicates a shift of high-risk people from children and young adolescents to the old, primarily the elevated hospitalization rates among the old people in Lianyungang (RR: 1.53, 95%CI: 1.46, 1.60) and Nantong (RR: 1.65, 95%CI: 1.57, 1.72) relative to those for children and young adolescents. Over the course of our study period, people with underlying diseases would have 26.5% (22.8–30.3%) and 12.7% (10.8–14.6%) higher odds of having longer hospitalisation and over 6 times higher odds of deaths after hospitalisation.

**Conclusions:**

Our estimates provide the first comprehensive evidence on the dynamic pollution-health associations throughout the pandemic. The results suggest that age and underlying diseases collectively determines the disparities of pollution-related health effect across population subgroups, underscoring the urgency to identifying the most vulnerable individuals to air pollution.

**Supplementary Information:**

The online version contains supplementary material available at 10.1186/s12940-024-01083-1.

## Background

Dynamics and burdens of respiratory diseases are shaped by interacting climatic and societal processes, including exposure to air pollution and human mobility pattern. Mounting evidence suggests that widespread non-pharmacal interventions during early pandemic quickly transformed social mixing and reduced pollution-attributable mortality [1–5]. To make robust assessment of such health benefits, there is a pressing need to critically analyse multifaceted health outcomes such as hospitalisations and mortality over a longer term.

Identifying the most vulnerable individuals to air pollution is a major public health concern. Given the age disparities in social activities and thus air pollution exposure, it is likely that air pollution may have disproportionately affected different age groups [6]. Recognizing the role of immunity, air pollution threatens to pose a dual burden to individuals with pre-existing diseases [7, 8]. This motivates effort to elucidate variations of pollution-related health outcomes across population subgroups. To date, many studies on respiratory diseases have reported findings on the association between ambient air pollution and hospital admissions and mortality [9–11]. Yet, heterogeneities across age groups and the overlapping burden for those with underlying diseases remain unknown.

The aim of this multicity study is to estimate age-stratified associations between air pollution exposure and health outcomes for respiratory diseases, such that recognizing potential changes between these associations during the COVID-19 pandemic. To this end, we first sought to quantify the health effects of air pollution exposure, both for hospital admissions and mortality. Additionally, we identified vulnerable population subgroups by assessing age differences in pollution-health associations and the excess risk of having underlying diseases.

## Methods

### Study population

Our study is based in 13 cities of Jiangsu province across a broad range of climatic conditions and sociodemographic structures (Supplemental Fig. 1). Individual-level records of respiratory diseases related-hospital admissions and mortality from Jan 1, 2017 to Dec 31, 2019 (defined as “pre-pandemic stage”) and from Jan 1, 2020 to Dec 31, 2022 (defined as “dynamic zero-COVID stage”) were retrieved from the surveillance system of Jiangsu Provincial Center for Disease Control and Prevention. The associated characteristics, including age, sex, postcode of residence, date of hospitalisation and mortality, diagnosis, and underlying diseases, were available for each individual. The underlying diseases considered include coronary heart disease, stroke, hypertension, chronic obstructive pulmonary disease, diabetes, chronic kidney disease, cancer, and immune defect.

### Health outcomes

Outcomes of interest differ between analyses on population and individual levels. At the population level, outcomes of interest included the daily number of hospital admissions and deaths for respiratory diseases. While a patient’s vulnerability was assessed by using the length of hospitalisations and death after hospitalisation. For simplicity, we categorized the patient’s length of hospital stay as less or longer than the median length among all individuals; and distinguished whether individuals died or recovered after hospitalisation.

### Air pollution exposure

We obtained daily air temperature at 2 m (m) and relative humidity from the fifth-generation reanalysis (ERA5) provided by the European Center for Medium-Range Weather Forecasts (ECMWF) [12]. Additionally, we collected daily 1 km (km) PM2.5 and 10 km O_3_ data from the Tracking Air Pollution in China (TAP) platform (http://tapdata.org.cn) which provides a near real-time distribution of air pollutants [13, 14]. By identifying the nearest grid point to the geographic location of individual’s residence, we further extracted the time-series data of local climate and pollution conditions.

### Statistical analyses

We conducted descriptive statistics for the city-stratified rate of hospitalisation and mortality for respiratory diseases. These rates were defined by the number of hospital admissions and deaths per 10,000 people. We quantified the changes of the rates by using the ratio of the rate in pre-pandemic and dynamic zero-COVID stages. All subsequent analyses that investigated potential changes in pollution-related health outcomes were done separately for the pre-pandemic and dynamic zero-COVID stages.

We started with evaluating the overall association between air pollution exposure and different health outcomes in an entire population. To do so, we used generalized additive model (GAM) to collate effects of pollution exposure and weather conditions on the rate of hospitalisations and mortality. Note that we focused on short-term effects of PM2.5 and O_3_ and assumed linear associations between pollutant predictors and health outcomes accordingly. We additionally included daily mean temperature and humidity as confounding factors to account for their non-linear effects on both hospitalisations and mortality. Therefore, two-pollutant models were established with the equation as follows:

1 $$\eqalign{{Y_{i,j}} = \,\, & {\alpha _{i,j}} + {\beta _1}P_{i - l1,j}^1 + {\beta _2}P_{i - l1,j}^2 + b\left( {{T_{i - l2,j}}} \right) \cr & + c\left( {R{H_{i - l2,j}}} \right) + {\gamma _j}{C_j} \cr}$$

where ${Y}_{i,j}$ is the number of hospital admissions or mortality on day $i$ in city $j$. The parameter ${\alpha }_{i,j}$ is the overall intercept. ${P}_{i-l1,j}^{1}$ and ${P}_{i-l1,j}^{2}$ is PM2.5 and O_3_ on single day, $l1$ (categorized as 0, 1, 2, or 3 days) prior to hospital admission or deaths, respectively; ${\beta }_{1}$ and ${\beta }_{2}$ are coefficients of two pollutant predictors respectively. $b\left({T}_{i-l2,j}\right)$ and $c\left({RH}_{i-l2,j}\right)$ is a smooth function accounting for daily mean temperature and humidity on cumulative days, $l2$, (categorized as 0–7, 0–14, 0–21, or 0–28 days) prior to the outcomes of interest, respectively. All models were adjusted for spatial heterogeneity by using a categorical variable to distinguish across cities, ${C}_{j}$. We fitted models by using records in the dynamic zero-COVID stage and used Generalized Cross-Validation (GCV) to determine the optimal lag effects. Assuming that these optimal lag effects were the same for all subsequent analyses, we further applied models to estimate pollution-health associations at the pre-pandemic stage.

Next, we extended the overall estimates to evaluate the age difference in the association between pollution factors and rate of hospitalisations and mortality. The age groups of the study population was children and young adolescents (aged 0–14 years old), working-age people (aged 15–64 years old), and the old population (over 65 years old). We considered records of hospital admissions among all three age groups; we restricted the analyses of mortality to working-age people and the old as deaths among children and young adolescents were very rare. GAMs were stratified by age groups and applied to each city using the following equation:

2 $$\eqalign{{Y_{i,a}} = \,\, & {\alpha _{i,a}} + {\beta _1}P_{i - lopt1,j}^1 + {\beta _2}P_{i - lopt1,j}^2 \cr & + b\left( {{T_{i - lopt2,j}}} \right) + c\left( {R{H_{i - lopt2,j}}} \right) + \gamma A \cr}$$

where ${Y}_{i,a}$ is the number of hospital admissions or mortality on day $i$ in age group $a$. The parameter ${\alpha }_{i,a}$ is the overall intercept. ${P}_{i-lopt1,j}^{1}$ and ${P}_{i-lopt1,j}^{2}$ are PM2.5 and O_3_ on the optimal time lag ($opt1$); while $b\left({T}_{i-lopt2,j}\right)$and $c\left({RH}_{i-lopt2,j}\right)$ are smooth functions for the multi-day lagged effect ($lopt2$) of temperature and humidity. Age groups are included as a categorical variable, $A$.

Further, we sought to assess the vulnerability of people with underlying diseases. We used multivariable logistic regression to examine the role of having underlying diseases on the odds of longer hospital stay and death after hospitalisation. Models were adjusted for confounding factors, including demographic characteristics (i.e. age and sex), geographic location of residence (rural or urban) and local weather conditions. We additionally accounted for the potential difference in the excess risk among individuals with different number of underlying diseases. We addressed this by evaluating the odds of hospitalisation and mortality for individuals with single disease as compared to those with double or more diseases.

### Sensitivity analyses

To validate our findings and insights, we first investigated variations in the pollution-health associations across age groups and cities. Furthermore, we examined potential uncertainty that may arise from the alternative classification of patients with underlying diseases. These individuals were segmented into three categories with single, double and multiple underlying diseases. Recognizing the control measures during the early pandemic and the potential modification of the pollution-related health effects, we make additional assessment of the association between pollutants and health outcomes in the dynamic zero-COVID stage by excluding the records in the first six months of 2020. All analyses were conducted with R version 4.3.0 and ArcGIS 10.8.1.

## Results

### Study population

**Fig. 1:**
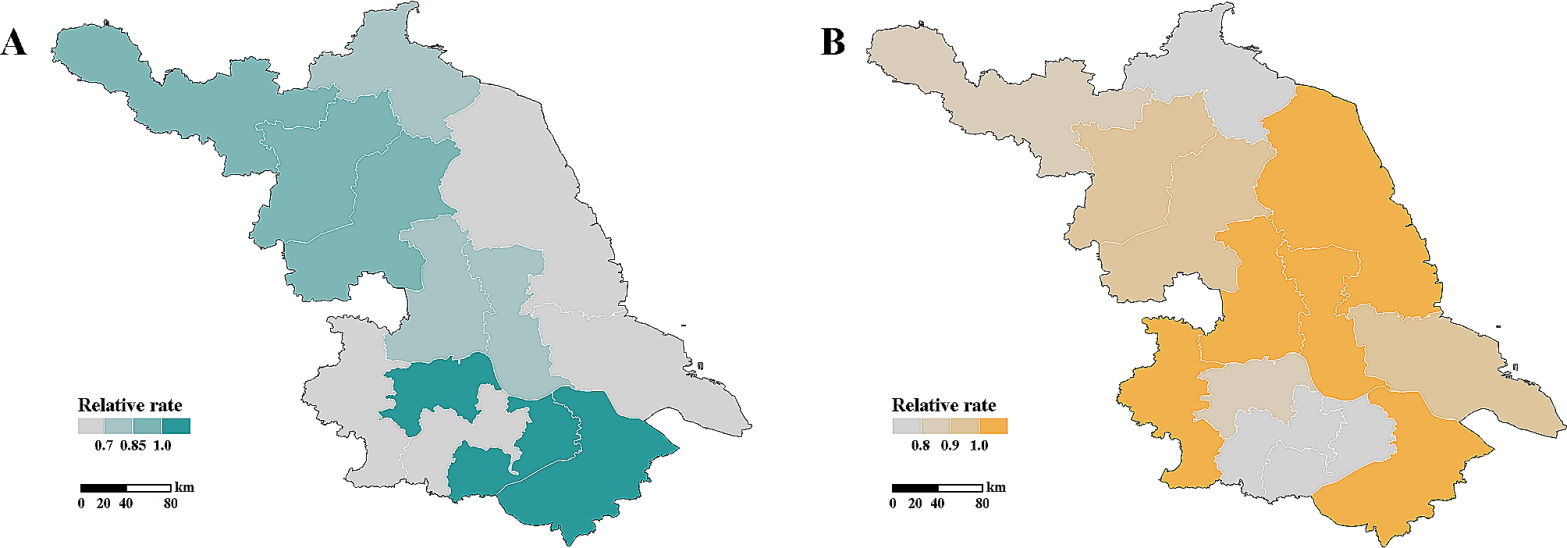
Hospital admissions and mortality for respiratory diseases across 13 cities in Jiangsu province. Cities are coloured by the relative rate of (**A**) hospitalisations and (**B**) mortality, i.e. the number of hospitalisations and death per 10,000, in pre-pandemic and dynamic zero-COVID stages

We retrieved 1,051,893 records of hospital admissions and 34,954 of mortality for respiratory disease during 2017–2022 in Jiangsu Province, China. The rate of hospitalisations and mortality differs significantly between the pre-pandemic and dynamic zero-COVID stages (Supplemental Fig. 2 and Supplemental Fig. 3); yet, spatial heterogeneity of such changes is observed (Fig. 1).

### Overall pollution-health associations

We first identified the overall association between air pollution exposure in the three days before hospital admission and hospitalisation rate for respiratory diseases (Table 1). For every 10 µg/m^3^ increase of PM2.5 and O_3_, the rate of hospital admission in the pre-pandemic stage increased by 0.2% (95% CI: 0.1–0.7%) and 0.7% (0.5–0.9%), respectively. Mortality of respiratory diseases correlates with pollutants exposure on the previous day (Table 1). It is estimated that the rate of mortality would increase by 1.8% (1.3–2.2%) with every 10 µg/m^3^ increase of O_3_.

**Table 1:** Assessment of the associations between pollutant exposure and rate of hospitalisation and mortality in pre-pandemic and dynamic zero-COVID stages

	Pre-pandemic stage	Dynamic zero-COVID stage
Hospitalisation rate		
PM2.5 (lag 3d)	1.002* (1.001,1.007)	1.014* (1.010, 1.017)
O_3_ (lag 3d)	1.007* (1.005,1.009)	0.983* (0.981, 0.985)
Mortality rate		
PM2.5 (lag 1d)	0.995 (0.989, 1.001)	0.996 (0.991, 1.003)
O_3_ (lag 1d)	1.018* (1.013, 1.022)	1.012* (1.006, 1.018)

Median and 95% credible intervals of the rate ratio is presented. Statistically significant results (*p* < 0.05) are marked with *

Statistically significant associations between pollutants and health outcomes are also seen in the dynamic zero-COVID stage. We found similar positive association between PM2.5 and hospitalisation rate, with a slightly higher effect (1.4% (1.0–1.7%) increase in hospitalisation rate) compared to pre-pandemic estimates. It is noted that the pattern of association between O_3_ and hospitalisations changes, yielding a 1.7% (1.5–1.9%) lower rate of hospitalisation for every 10 µg/m^3^ increase of O_3_. Additionally, we observed significant association between O_3_ and mortality. It shows that O_3_-related increase in the mortality rate would be lowered to 1.2% (0.6–1.8%) in the dynamic zero-COVID stage. Restricting analyses to subpopulations and geographic areas yield greater variations of the associations among age groups and cities (Supplemental Tables 1–2).

### Age difference in pollution-health associations

**Fig. 2:**
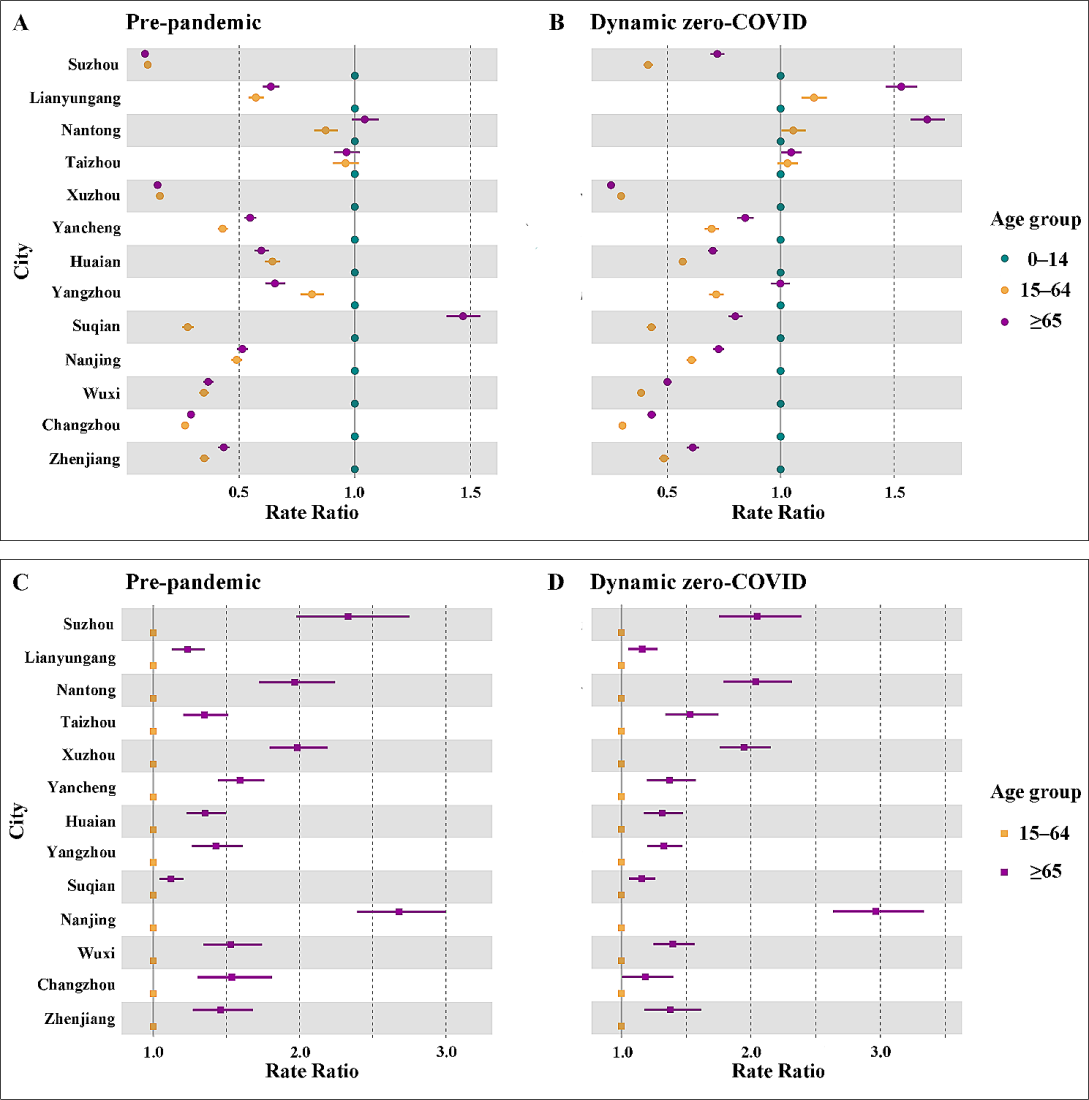
Age disparities in pollution-related rate of hospitalisation and mortality. Estimates of (**A**–**B**) hospitalisation and (**C**–**D**) mortality rate across age groups in pre-pandemic and dynamic zero-COVID stage are presented

Disaggregation of age groups indicates heterogeneity in pollution-health associations within the population (Fig. 2). Prior to the COVID-19 pandemic, children and young adolescents were at the highest rate of hospitalisation for respiratory diseases across cities, except for Suqian (Fig. 2A). However, such age-stratified associations between pollution and hospital admission shifted in the dynamic zero-COVID stage (Fig. 2B), with substantially elevated rates observed for the old people in Lianyungang (RR: 1.53, 95%CI: 1.46, 1.60) and Nantong (RR: 1.65, 95%CI: 1.57, 1.72) relative to those for children and young adolescents. This increase generates a shift of high-risk people from children and young adolescents to the old. Conversely, we identify that children and the young adolescents turn into the high-risk group with the highest rate of hospitalisation in Suqian (Fig. 2B). Limiting associations to individuals over 14 years shows that mortality rates are generally greater among the old population, with RR values ranging from 1.12 to 2.96, as compared to those for the working-age people across cities and pandemic stages (Fig. 2C–D).

### Excess risk of having underlying diseases

After adjusting for age, sex, and residence, patients having underlying diseases correlate with significantly increased odds of longer hospital stay and death after hospitalisation (Table 2). It is estimated that the medium length of hospitalisation over the study period is seven days. Compared with this, our estimates show that people with underlying diseases would have 26.5% (22.8–30.3%) more odds of having longer hospitalisation than those without underlying diseases. Such increase of the odds attributable to underlying diseases are slightly attenuated to 12.7% (10.8–14.6%) in the dynamic zero-COVID stage. Of note, patients with underlying diseases have a strikingly higher odds of deaths after hospitalisation. Over the course of our study period, these patients have odds of deaths that are over 6 times (6.4 (5.6, 7.3) in the pre-pandemic stage and 6.1 (5.7, 6.6) in the dynamic zero-COVID stage) than those in the reference group.

**Table 2:** Odds ratio of the overlapping burden among individuals with underlying diseases

	Pre-pandemic stage		Dynamic zero-COVID stage	
	Hospitalisation OR (95%CI)	Mortality OR (95%CI)	Hospitalisation OR (95%CI)	Mortality OR (95%CI)
Age, years	1.015* (1.014, 1.015)	1.085* (1.139, 1.337)	1.019* (1.019, 1.020)	1.084* (1.082, 1.087)
Sex				
Female	1 (ref)	1 (ref)	1 (ref)	1 (ref)
Male	1.086* (1.062, 1.110)	1.233* (1.139, 1.337)	1.066* (1.051, 1.080)	1.161* (1.108, 1.218)
Underlying diseases				
No	1 (ref)	1 (ref)	1 (ref)	1 (ref)
Yes	1.265* (1.228, 1.303)	6.376* (5.578, 7.326)	1.127* (1.108, 1.146)	6.136* (5.698, 6.618)
Residential				
Rural	1 (ref)	1 (ref)	1 (ref)	1 (ref)
Urban	1.149* (1.123, 1.175)	0.362* (0.336, 0.391)	1.033* (1.019, 1.047)	0.521* (0.499, 0.544)
PM2.5, 10 µg/m^3^	0.994* (0.989, 0.999)	0.989 (0.974, 1.004)	1.007* (1.005, 1.010)	0.971* (0.961, 0.980)
O_3_, 10 µg/m^3^	1.015* (1.012, 1.018)	0.952* (0.943, 0.962)	0.994* (0.992, 0.996)	1.012* (1.006, 1.018)
Temperature, ˚C	0.994* (0.989, 0.993)	1.002 (0.996, 1.008)	0.998* (0.997, 0.998)	0.979* (0.977, 0.982)
Humidity, %	1.005* (1.004, 1.007)	0.998 (0.993, 1.002)	1.006* (1.005, 1.007)	1.004* (1.001, 1.007)

Statistically significant results (*p* < 0.05) are marked with *

Further assessment indicates disparities in the odds of deaths among patients with single and multiple underlying diseases (Supplemental Table 3). Compared to patients without underlying disease, our estimates show that having single underlying disease is associated with around 20% and 540% higher odds of longer hospital stay and death after hospitalisation, respectively. These estimates surge, primarily the odds of deaths in the dynamic zero-COVID stage, among those with double or more underlying diseases. Sensitivity analyses confirm our finding of the increased odds of hospitalisation and death attributable to underlying diseases (Supplemental Table 4).

## Discussion

Our multicity study fits well in the current context of the exacerbated health effects of air pollution exposure. In light of emerging evidence that pollution-attributable mortality may have reduced during the pandemic [1–5], we provided the first essential evidence on the dynamic pollution-health associations throughout the pandemic. Unlike previous studies focusing solely on mortality, our multicity study in Jiangsu province extends the analysis to include hospitalizations, thereby offering a more multifaceted understanding how pandemic-related interventions may have modulated the influence of pollutants on health outcomes. We found that air pollution exposure generally associates with multifaceted health outcomes, including hospitalisations and mortality, for respiratory diseases. This finding is broadly consistent with other studies done across a variety of settings [15–21]. It is important to note that, over time, there is an opposite shift in the pattern of association between air pollutants and hospitalisation rates. The effect of O_3_ on the hospitalisation rate was attenuated which, in turn, may be partially attributed to the lowered level of O_3_ during the pandemic. In contrast, risk of PM2.5-related hospitalisation increased in the dynamic zero-COVID stage. Although associations between pollutants and hospital admission evolves, we detected no evidence of deviations from the pre-pandemic association between pollutants exposure and mortality.

When considering the disparities of pollution-related health outcomes among population subgroups, our findings emphasize the importance of two main axes of vulnerability to air pollution exposure: age and underlying diseases. Over the course of the pandemic, the shift in the age profile of high-risk groups may be largely dependent on the relative fraction of hospitalisation across age groups (Supplemental Fig. 3). Specifically, the fraction of hospitalisation for respiratory diseases increased among children and young adolescents but reduced among the old in Suqian. Such trade-off generated the shift of the high-risk group from the old to children and young adolescents. Similarly, people over 65 accounted for the greatest fraction of hospitalisation in Lianyungang and Nantong in the dynamic zero-COVID stage, resulting in high-risk population shifts from children and young adolescents to the elderly. Moving beyond the age burden, estimates of an individual’s excess risk would additionally heighten the overlapping burden from air pollution and underlying diseases.

Despite the clear signature of age and underlying diseases on individual’s vulnerability, we provided essential evidence that sex and location of residence are strongly associated with the increased odds of longer hospitalisation and higher risk of death (Table 2). We identified that male is more likely to have a higher risk of longer hospital admission (8.6% (95% CI 6.2–11%) in pre-pandemic and 6.6% (95% CI 5.1–8.0%) in dynamic zero-COVID stage) relative to females. Similarly, males tend to have 23.3% (13.9–33.7%) and 16.1% (10.8–21.8%) higher risk of death after hospitalisation in two pandemic stages, respectively. This finding is similar to other environmental health studies, highlighting gender difference in the risk of air pollution exposure [22–26]. Additionally, living in urban areas would associate with a 14.9% (12.3–17.5%) increase in the risk of longer hospitalisation but a 63.8% (60.9–66.4%) lowered mortality risk prior to the pandemic. Although this urban-rural difference is narrowed in the dynamic zero-COVID stage, it is mostly attributed to the health inequalities among areas [27]. Inadequate health resources yield the limited access to health facilities for people living in rural areas. In such context, people may not seek care in health facilities, leading to a great reduction of hospital admission and the lowered risk of hospitalisation accordingly. With respect to mortality rate, the limited health resources may delay the treatment of respiratory diseases and therefore contribute to higher odds of death after hospitalisation for the rural population.

The key messages of this study are of great public health significance. We provided fundamental estimates of the dynamic pollution-health associations over time, which makes critical contributions to understanding how pandemic-related interventions may have modulated the influence of pollutants on health outcomes. Importantly, by identifying vulnerable population subgroups, particularly the elderly and those with preexisting conditions, we underscore the disproportionate effects of air pollution. These findings support WHO global air quality guidelines where the elderly and those with underlying diseases bear the greatest burden of air pollution [28]. Additionally, this study based on a multicity approach across 13 cities in Jiangsu province, offers a comprehensive perspective that enhances the generalizability of our findings. In light of these evidence, health authorities will build a complete picture of the heterogeneous health effects of air pollution exposure for respiratory diseases, formulating relevant policies to identify and protect the most vulnerable individuals.

Future studies may build on our findings in several ways. First, we have explicitly estimated air pollution effects on hospitalisation and mortality. Examining across a variety of pollutants and outcomes is essential for differentiating the roles of co-occurring air pollutants on respiratory outcomes. Additionally, assessment of the pollution-related health effects may vary by applying alternative statistical approaches. Evaluating the potential bias of assessment attributable to the statistical approach would be a promising direction for future investigations. Furthermore, our inability to obtain hospitalisation records in 2017–2018 is likely to bias our observed associations which should be validated. Finally, we have reported the excess risk of having underlying diseases. Moving from this finding, prioritizing the most vulnerable subpopulation will require distinguishing contributions across specific underlying diseases.

### Electronic supplementary material

Below is the link to the electronic supplementary material.

Supplementary Material 1

## Data Availability

All data were archived within the surveillance system of the Jiangsu Provincial Center for Disease Control and Prevention. The datasets used and/or analysed during the current study are available from the corresponding author on reasonable request.

## Abbreviations

ERA5: Fifth-generation reanalysis
ECMWF: European Center for Medium-Range Weather Forecasts
TAP: Tracking Air Pollution in China
GAM: Generalized additive model

## References

[1] Chen K, Wang M, Huang C, Kinney PL, Anastas PT (2020). Air pollution reduction and mortality benefit during the COVID-19 outbreak in China. Lancet Planet Health.

[2] Giani P, Castruccio S, Anav A, Howard D, Hu W, Crippa P (2020). Short-term and long-term health impacts of air pollution reductions from COVID-19 lockdowns in China and Europe: a modelling study. Lancet Planet Health.

[3] Hao X, Li J, Wang H, Liao H, Yin Z, Hu JF (2021). Long-term health impact of PM2.5 under whole-year COVID-19 lockdown in China. Environ Pollut.

[4] Chen G, Tao J, Wang J, Dong M, Li X, Sun X (2021). Reduction of air pollutants and associated mortality during and after the COVID-19 lockdown in China: impacts and implications. Environ Res.

[5] Venter ZS, Aunan K, Chowdhury S, Lelieveld J (2020). Air pollution declines during COVID-19 lockdowns mitigate the global health burden. Environ Res.

[6] Cai W, Zhang C, Zhang S, Bai Y, Callaghan M, Chang N (2022). The 2022 China report of the Lancet countdown on health and climate change: leveraging climate actions for healthy ageing. Lancet Public Health.

[7] Hooper LG, Kaufman JD (2018). Ambient air Pollution and clinical implications for susceptible populations. Ann Am Thorac Soc.

[8] Tibuakuu M, Michos ED, Navas-Acien A, Jones MR (2018). Air Pollution and Cardiovascular Disease: a focus on vulnerable populations Worldwide. Curr Epidemiol Rep.

[9] Lin C, Ma Y, Liu R, Shao Y, Ma Z, Zhou L (2022). Associations between short-term ambient ozone exposure and cause-specific mortality in rural and urban areas of Jiangsu, China. Environ Res.

[10] Ma Y, Zhou L, Chen K (2020). Burden of cause-specific mortality attributable to heat and cold: a multicity time-series study in Jiangsu Province, China. Environ Int.

[11] Wang C, Feng L, Chen K (2019). The impact of ambient particulate matter on hospital outpatient visits for respiratory and circulatory system disease in an urban Chinese population. Sci Total Environ.

[12] Hersbach H, Bell B, Berrisford P, Hirahara S, Horányi A, Muñoz-Sabater J (2020). The ERA5 global reanalysis. Q J Roy Meteor Soc.

[13] Geng XQ, Liu G, Liu S, Meng J, Zhang X (2022). Spatiotemporal continuous estimates of daily 1 km PM_2.5_ from 2000 to present under the Tracking Air Pollution in China (TAP) framework. Atmos Chem Phys.

[14] Xue T, Zheng Y, Geng G, Xiao Q, Meng X, Wang M (2020). Estimating Spatiotemporal variation in ambient ozone exposure during 2013–2017 using a Data-Fusion Model. Environ Sci Technol.

[15] Lu P, Zhang Y, Lin J, Xia G, Zhang W, Knibbs LD (2020). Multi-city study on air pollution and hospital outpatient visits for asthma in China. Environ Pollut.

[16] Zhang Y, Wu Z, Gou K, Wang R, Wang J (2021). The impact of Air Pollution on Outpatient visits of children with asthma in Xi’an, China. Wild Environ Med.

[17] Gu J, Shi Y, Zhu Y, Chen N, Wang H, Zhang Z (2020). Ambient air pollution and cause-specific risk of hospital admission in China: a nationwide time-series study. PLoS Med.

[18] Wang M, Chen J, Zhang Z, Yu P, Gan W, Tan Z (2020). Associations between air pollution and outpatient visits for arrhythmia in Hangzhou, China. BMC Public Health.

[19] Yin P, Chen R, Wang L, Meng X, Liu C, Niu Y (2017). Ambient ozone Pollution and Daily Mortality: a nationwide study in 272 Chinese cities. Environ Health Persp.

[20] Perone G (2022). Assessing the impact of long-term exposure to nine outdoor air pollutants on COVID-19 spatial spread and related mortality in 107 Italian provinces. Sci Rep.

[21] Orellano P, Reynoso J, Quaranta N, Bardach A, Ciapponi A (2020). Short-term exposure to particulate matter (PM10 and PM2.5), nitrogen dioxide (NO2), and ozone (O3) and all-cause and cause-specific mortality: systematic review and meta-analysis. Environ Int.

[22] Ren M, Li N, Wang Z, Liu Y, Chen X, Chu Y (2017). The short-term effects of air pollutants on respiratory disease mortality in Wuhan, China: comparison of time-series and case-crossover analyses. Sci Rep.

[23] Li M, Chen S, Zhao H, Tang C, Lai Y, Oi C (2021). The short-term associations of chronic obstructive pulmonary disease hospitalizations with meteorological factors and air pollutants in Southwest China: a time-series study. Sci Rep.

[24] Shin HH, Parajuli RP, Gogna P, Maquiling A, Dehghani P (2020). Pollutant-sex specific differences in respiratory hospitalization and mortality risk attributable to short-term exposure to ambient air pollution. Sci Total Environ.

[25] Niu Y, Zhou Y, Chen R, Yin P, Meng X, Wang W (2022). Long-term exposure to ozone and cardiovascular mortality in China: a nationwide cohort study. Lancet Planet Health.

[26] Ma Z, Meng X, Chen C, Chao B, Zhang C, Li W (2022). Short-term effects of different PM2.5 ranges on daily all-cause mortality in Jinan, China. Sci Rep.

[27] Zhang X, Dupre ME, Qiu L, Zhou W, Zhao Y, Gu D (2017). Urban-rural differences in the association between access to healthcare and health outcomes among older adults in China. BMC Geriatr.

[28] WHO global air quality guidelines. Particulate matter (PM2.5 and PM10), ozone, nitrogen dioxide, sulfur dioxide and carbon monoxide. Geneva: World Health Organization; 2021. 34662007

