# Supplementary material for: The changing health effects of air pollution exposure for respiratory diseases: a multicity study during 2017–2022
Source: Environ Health. 2024 Apr 13;23:36. doi: 10.1186/s12940-024-01083-1 (PMC11015632; doi:10.1186/s12940-024-01083-1)
Supplement: Supplementary file 1 — Supplementary Material 1 [file 12940_2024_1083_MOESM1_ESM.docx]

***Supplementary Material for***

**The changing health effects of air pollution exposure for respiratory diseases: a multicity study during 2017–2022**

***Environmental Health***

Siyu Jiang^1†^, Longjuan Tang^1†^, Zhe Lou^1†^, Haowei Wang^2,3^, Ling Huang^4^, Wei Zhao^1^, Qingqing Wang^5^, Ruiyun Li^1*^, Zhen Ding^5*^

^1^School of Public Health, Nanjing Medical University, Nanjing, Jiangsu, China

^2^School of Public Health, Imperial College London, UK

^3^MRC Centre for Global Infectious Disease Analysis and Abdul Latif Jameel Institute for Disease and Emergency Analytics, Imperial College London, UK

^4^College of Urban and Environmental Sciences, Peking University, Beijing, China

^5^Jiangsu Provincial Center for Disease Prevention and Control, Nanjing, China

^†^These authors contributed equally to this work.

^*^Correspondence to:

Z.D.: Email: jscdc@126.com; Tel.: +86 18915999356; Address: Jiangsu Provincial Center for Disease Prevention and Control, 172 Jiangsu Rd, Nanjing, Jiangsu, 210009, China

R.L.: Email: ruiyun.li@njmu.edu.cn; Tel.: +86 18811473393; Address: School of Public Health, Nanjing Medical University, 101 Longmian AV., Nanjing, Jiangsu, 211166, China

**Supplemental Fig. 1 Climatic and demographic features of the cities in Jiangsu province, China.** City-level monthly mean (A) temperature and (B) humidity over 2017–2022 are presented. (C) The age structure is characterized by the proportion of individuals aged 0–14, 15–64 and over 65 years old.

**Supplemental Fig. 2 Hospitalisation and mortality rate for respiratory diseases in pre-pandemic and dynamic zero-COVID stages.** The rate of (A) hospitalisation (or the number of hospital admissions per 10,000 individuals) and (B) mortality (or the number of mortality per 100,000 individuals) are presented separately for pre-pandemic and dynamic zero-COVID stages. Statistical analyses of the rates between two stages are marked.

**Supplemental Fig. 3 City-level hospitalisation and mortality rate for respiratory diseases in pre-pandemic and dynamic zero-COVID stages.** The rate of (A) hospitalisation and (B) mortality, or the number of hospital admissions and mortality per 10,000 individuals, in each city are presented separately for pre-pandemic and dynamic zero-COVID stages.

**Supplemental Fig. 4 Relative fraction of hospitalisation across age groups in pre-pandemic and dynamic zero-COVID stages.**

**Supplemental Table 1. The age and spatial differences in the associations between pollutant exposure and rate of hospitalisation and mortality in pre-pandemic stage.** Median and 95% credible intervals of the rate ratio is presented. Statistically significant results (p<0.05) are marked with *.

|  | Hospitalisation | | Mortality | |
| --- | --- | --- | --- | --- |
|  | PM2.5 (lag 3d) | O_3_ (lag 3d) | PM2.5 (lag 1d) | O_3_ (lag 1d) |
| Age group, years | | | | |
| 0-14 | 1.005* (1.001, 1.009) | 0.999 (0.997, 1.001) | 1.033 (0.994, 1.073) | 0.998 (0.963, 1.033) |
| 15-64 | 0.992* (0.986, 0.999) | 1.016* (1.012,1.019) | 0.998 (0.981, 1.015) | 1.025* (1.010, 1.039) |
| ≥65 | 0.993* (0.988, 0.999) | 1.021* (1.017, 1.024) | 0.994 (0.987, 1.000) | 1.017* (1.012, 1.022) |
| City |  |  |  |  |
| Lianyungang | 0.998 (0.989, 1.007) | 1.011* (1.004, 1.017) | 1.004 (0.991, 1.017) | 1.006 (0.996, 1.017) |
| Xuzhou | 1.016* (1.009, 1.023) | 0.982* (0.977, 0.987) | 1.004 (0.994, 1.013) | 1.008 (0.999, 1.017) |
| Suqian | 0.999 (0.991, 1.008) | 1.007* (1.001, 1.015) | 0.990 (0.979, 1.001) | 0.997 (0.987, 1.007) |
| Huaian | 0.982* (0.973, 0.992) | 1.026* (1.019, 1.033) | 1.000 (0.987, 1.013) | 1.002 (0.990, 1.015) |
| Yancheng | 1.005 (0.994, 1.015) | 1.025* (1.015, 1.035) | 0.997 (0.984, 1.010) | 1.010 (0.992, 1.028) |
| Taizhou | 0.993 (0.981, 1.005) | 1.012* (1.004, 1.019) | 1.000 (0.986, 1.014) | 1.006 (0.994, 1.018) |
| Yangzhou | 0.974* (0.961, 0.987) | 1.025* (1.017, 1.033) | 0.980* (0.965, 0.996) | 1.022* (1.009, 1.035) |
| Nanjing | 0.993 (0.982, 1.004) | 1.009* (1.004, 1.014) | 0.993 (0.979, 1.006) | 1.006 (0.997, 1.015) |
| Nantong | 1.009 (0.998, 1.020) | 1.006* (1.000, 1.013) | 0.991 (0.976, 1.006) | 1.008 (0.999, 1.017) |
| Changzhou | 0.988* (0.978, 0.997) | 1.009* (1.004, 1.014) | 1.007 (0.990, 1.024) | 1.021* (1.008, 1.035) |
| Wuxi | 0.983* (0.969, 0.996) | 1.013* (1.005, 1.022) | 1.013 (0.999, 1.028) | 1.004 (0.995, 1.014) |
| Suzhou | 1.002 (0.993, 1.012) | 1.001 (0.997, 1.006) | 0.997 (0.982, 1.012) | 1.011 (0.999, 1.023) |
| Zhenjiang | 0.989 (0.978, 1.001) | 1.013* (1.008, 1.019) | 0.984* (0.969, 0.999) | 1.018* (1.007, 1.029) |

**Supplemental Table 2. The age and spatial differences in the associations between pollutant exposure and rate of hospitalisation and mortality in dynamic zero-COVID stage.** Median and 95% credible intervals of the rate ratio is presented. Statistically significant results (p<0.05) are marked with *.

|  | Hospitalisation | | Mortality | |
| --- | --- | --- | --- | --- |
|  | PM2.5 (lag 3d) | O_3_ (lag 3d) | PM2.5 (lag 1d) | O_3_ (lag 1d) |
| Age group, years | | | | |
| 0-14 | 1.025* (1.021, 1.030) | 0.968* (0.965, 0.971) | 0.952 (0.869, 1.044) | 1.033 (0.957, 1.116) |
| 15-64 | 1.000 (0.996, 1.005) | 0.995* (0.992,0.998) | 1.007 (0.981, 1.034) | 1.013 (0.994, 1.033) |
| ≥65 | 1.000 (0.996, 1.004) | 0.998 (0.996, 1.001) | 1.004 (0.995, 1.013) | 1.012* (1.005, 1.018) |
| City |  |  |  |  |
| Lianyungang | 1.019* (1.009, 1.028) | 0.980* (0.973, 0.986) | 0.992 (0.976, 1.008) | 1.001 (0.988, 1.013) |
| Xuzhou | 1.015* (1.007, 1.023) | 0.980* (0.972, 0.987) | 1.005 (0.991, 1.019) | 1.023* (1.010, 1.036) |
| Suqian | 1.009* (1.000, 1.017) | 0.989* (0.981, 0.996) | 0.999 (0.984, 1.014) | 1.002 (0.988, 1.016) |
| Huaian | 1.005 (0.998, 1.013) | 0.997 (0.992, 1.002) | 1.006 (0.986, 1.027) | 0.999 (0.985, 1.013) |
| Yancheng | 1.001 (0.990, 1.012) | 0.997 (0.990, 1.005) | 1.017 (0.991, 1.044) | 1.023* (1.002, 1.045) |
| Taizhou | 1.011* (1.000, 1.021) | 0.995 (0.989, 1.001) | 1.024* (1.003, 1.047) | 1.002 (0.988, 1.017) |
| Yangzhou | 1.014* (1.004, 1.025) | 0.991* (0.985, 0.997) | 1.012 (0.993, 1.031) | 1.010 (0.996, 1.023) |
| Nanjing | 1.011* (1.003, 1.019) | 0.983* (0.979, 0.987) | 0.999 (0.983, 1.015) | 1.005 (0.995, 1.016) |
| Nantong | 1.005 (0.993, 1.017) | 0.990* (0.983, 0.997) | 0.995 (0.976, 1.015) | 1.000 (0.987, 1.013) |
| Changzhou | 1.023* (1.013, 1.033) | 0.974* (0.968, 0.979) | 0.989 (0.964, 1.015) | 1.020* (1.003, 1.038) |
| Wuxi | 1.024* (1.015, 1.033) | 0.975* (0.970, 0.980) | 1.016 (0.999, 1.033) | 1.000 (0.989, 1.011) |
| Suzhou | 1.019* (1.008, 1.031) | 0.975* (0.969, 0.980) | 0.997 (0.978, 1.017) | 1.003 (0.991, 1.015) |
| Zhenjiang | 1.017* (1.006, 1.029) | 0.982* (0.976, 0.988) | 0.975* (0.954, 0.997) | 1.011 (0.997, 1.025) |

**Supplemental Table 3. Odds ratio of the overlapping burden among individuals with underlying diseases.** Individuals were segmented into two categories with single and double or more underlying diseases. Statistically significant results (p<0.05) are marked with *.

|  | Pre-pandemic stage | | Dynamic zero-COVID stage | |
| --- | --- | --- | --- | --- |
|  | Hospitalisation  OR (95%CI) | Mortality  OR (95%CI) | Hospitalisation  OR (95%CI) | Mortality  OR (95%CI) |
| Age, years | 1.015* (1.014, 1.015) | 1.085* (1.081, 1.089) | 1.019* (1.019, 1.020) | 1.084* (1.081, 1.086) |
| Sex |  |  |  |  |
| Female | 1 (ref) | 1 (ref) | 1 (ref) | 1 (ref) |
| Male | 1.085* (1.061, 1.109) | 1.234* (1.139, 1.337) | 1.063* (1.049, 1.078) | 1.156* (1.104, 1.211) |
| Number of underlying diseases | | | | |
| 0 | 1 (ref) | 1 (ref) | 1 (ref) | 1 (ref) |
| 1 | 1.246* (1.209, 1.282) | 6.442* (5.631, 7.405) | 1.112* (1.093, 1.131) | 6.414* (5.954, 6.920) |
| ≥2 | 1.492* (1.396,1.595) | 5.899* (4.975, 7.008) | 1.284* (1.232,1.340) | 8.214* (7.512, 8.990) |
| Residential |  |  |  |  |
| Rural | 1 (ref) | 1 (ref) | 1 (ref) | 1 (ref) |
| Urban | 1.144* (1.119, 1.170) | 0.364* (0.337, 0.392) | 1.035* (1.021, 1.048) | 0.522* (0.500, 0.545) |
| PM2.5, 10μg/m^3^ | 0.995* (0.990, 0.999) | 0.989 (0.974, 1.004) | 1.007* (1.005, 1.010) | 0.971* (0.961, 0.980) |
| O_3_, 10μg/m^3^ | 1.015* (1.012, 1.018) | 0.953* (0.943, 0.962) | 0.994* (0.992, 0.996) | 1.010* (1.004, 1.016) |
| Temperature, ˚C | 0.994* (0.992, 0.996) | 1.002 (0.996, 1.008) | 0.998* (0.997, 0.998) | 0.979* (0.977, 0.982) |
| Humidity, % | 1.005* (1.003, 1.006) | 0.998 (0.993, 1.002) | 1.006* (1.005, 1.007) | 1.005* (1.002, 1.007) |

**Supplemental Table 4. Odds ratio of the overlapping burden among individuals with underlying diseases.** Individuals were segmented into three categories with single, double and multiple underlying diseases.

|  | Pre-pandemic stage | | Dynamic zero-COVID stage | |
| --- | --- | --- | --- | --- |
|  | Hospitalisation  OR (95%CI) | Mortality  OR (95%CI) | Hospitalisation  OR (95%CI) | Mortality  OR (95%CI) |
| Age, years | 1.015* (1.014, 1.015) | 1.085* (1.081, 1.089) | 1.019* (1.019, 1.020) | 1.084* (1.081, 1.086) |
| Sex |  |  |  |  |
| Female | 1 (ref) | 1 (ref) | 1 (ref) | 1 (ref) |
| Male | 1.085* (1.061, 1.109) | 1.234 (1.140, 1.338) | 1.063* (1.049, 1.078) | 1.155* (1.103, 1.210) |
| Number of underlying diseases | | | | |
| 0 | 1 (ref) | 1 (ref) | 1 (ref) | 1 (ref) |
| 1 | 1.246* (1.209, 1.284) | 6.440* (5.630, 7.403) | 1.112* (1.093, 1.131) | 6.413* (5.953, 6.919) |
| 2 | 1.473* (1.373, 1.583) | 6.404* (5.381, 7.632) | 1.260* (1.206, 1.317) | 8.427* (7.693, 9.237) |
| ≥3 | 1.604* (1.363, 1.899) | 3.220* (2.150, 4.661) | 1.570* (1.368, 1.808) | 6.597* (5.459, 7.926) |
| Residential |  |  |  |  |
| Rural | 1 (ref) | 1 (ref) | 1 (ref) | 1 (ref) |
| Urban | 1.144* (1.119, 1.170) | 0.364* (0.338, 0.393) | 1.035* (1.021, 1.048) | 0.522* (0.500, 0.545) |
| PM2.5, 10μg/m^3^ | 0.995* (0.990, 0.999) | 0.988 (0.973, 1.004) | 1.007* (1.005, 1.010) | 0.971* (0.962, 0.980) |
| O_3_, 10μg/m^3^ | 1.015* (1.012, 1.017) | 0.953* (0.943, 0.963) | 0.994* (0.992, 0.996) | 1.010* (1.004, 1.016) |
| Temperature, ˚C | 0.994* (0.992, 0.996) | 1.002 (0.996, 1.008) | 0.998* (0.997, 0.998) | 0.980* (0.977, 0.982) |
| Humidity, % | 1.005* (1.003, 1.006) | 0.998 (0.993, 1.002) | 1.006* (1.005, 1.007) | 1.005* (1.002, 1.007) |

**Supplemental Table 5. Assessment of the associations between pollutant exposure and rate of hospitalisation and mortality in pre-pandemic and post-pandemic stages.** Health records in the first six month of 2020 were excluded from the assessment for the dynamic zero-COVID stage.

|  | | Pre-pandemic stage | | Dynamic zero-COVID stage | |
| --- | --- | --- | --- | --- | --- |
| Hospitalisation rate | |  | |  | |
| PM2.5 (lag 3d) | | 1.002* (1.001,1.007) | | 1.006* (1.003, 1.009) | |
| O_3_ (lag 3d) | | 1.007* (1.005,1.009) | | 0.993* (0.991, 0.996) | |
| Mortality rate | |  | |  | |
| PM2.5 (lag 1d) | | 0.995 (0.989, 1.001) | | 1.002 (0.993, 1.011) | |
| O_3_ (lag 1d) | | 1.018* (1.013, 1.022) | | 1.006* (1.001, 1.012) | |

Median and 95% credible intervals of the rate ratio is presented. Statistically significant results (p<0.05) are marked with *.
